# Endoscopic ultrasound-guided vascular intervention for pancreaticojejunal variceal bleeding

**DOI:** 10.1055/a-2291-9619

**Published:** 2024-04-09

**Authors:** Nozomi Okuno, Kazuo Hara, Shin Haba, Takamichi Kuwahara, Minako Urata, Takashi Kondo, Yoshitaro Yamamoto

**Affiliations:** 1 538363 Gastroenterology, Aichi Cancer Center Hospital, Nagoya, Japan

Ectopic variceal bleeding is rare, only accounting for up to 5% of all variceal bleeding 1 ; however, a previous study reported a higher risk of bleeding and mortality for ectopic varices than for esophageal varices 2 . Endoscopic treatment can be difficult because of difficulty in identifying the location of the bleeding 3 . Endoscopic ultrasound (EUS)-guided vascular intervention has recently been reported to be particularly useful in situations where traditional approaches may be challenging or ineffective 4 .

A man in his 70s was admitted to our hospital for the third time with tarry stools and anemia. He had undergone pancreaticoduodenectomy for intraductal papillary mucinous neoplasm 3 years previously. It was possible to reach the site of the pancreaticojejunostomy with a colonoscope and pancreaticojejunal varices were detected. Twice previously, the patient had undergone endoscopic clipping and had received a blood transfusion before being discharged; however, he was readmitted within 1.5 years, at which time a computed tomography (CT) scan and endoscopy revealed pancreaticojejunal varices ( Fig. 1 ). Because of his recurrent bleeding, we planned to perform EUS-guided vascular intervention ( Video 1 ).

**Fig. 1:**
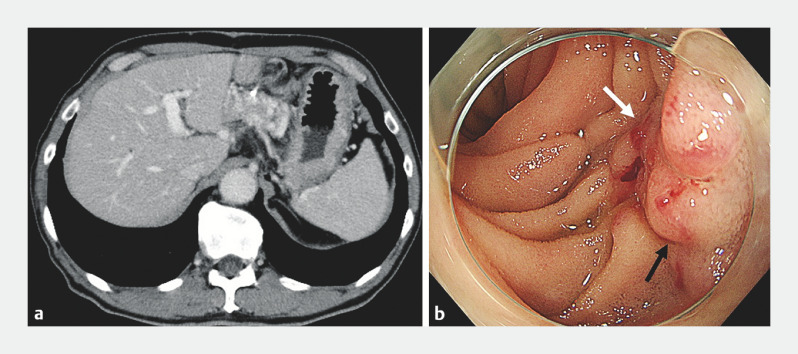
Pancreaticojejunal varices are seen on: **a** computed tomography, with varices evident near the site of the pancreaticojejunostomy; **b** endoscopy, with visible blood vessels (white arrowhead) and nodular vascular dilatations (black arrowhead) at the pancreaticojejunal anastomosis.

Endoscopic ultrasound-guided vascular intervention is performed for the treatment of recurrent pancreaticojejunal variceal bleeding.Video 1

We changed the scope to a forward-viewing linear echoendoscope (TGF-UC260 J; Olympus, Tokyo, Japan), which was passed through the afferent jejunal limb. EUS was used to detect the pancreaticojejunal varices using color Doppler. We punctured the varices with a 19G needle (EZ shot 3 plus; Olympus) before injecting a mixture of 1.5 mL of Histoacryl and 0.5 mL of Lipiodol (2 mL in total). Post-treatment fluoroscopy confirmed successful occlusion of the varices ( Fig. 2 **a** ). Plain CT performed on the following day also showed complete variceal occlusion ( Fig. 2 **b** ). No adverse events, such as pancreatitis, were observed, and the patient was discharged on the fourth day after the procedure. Follow-up endoscopy, 1 year later, revealed obliterated varices ( Fig. 3 ). No further bleeding has occurred in more than 1.5 years.

**Fig. 2:**
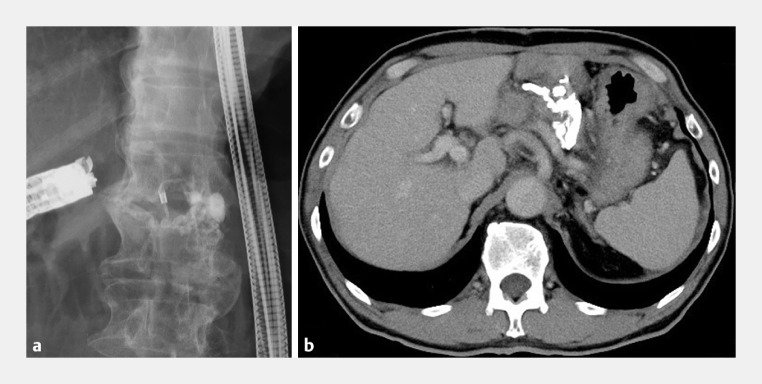
Complete occlusion of the varices after injection with a mixture of Histoacryl and Lipiodol is shown on: **a** fluoroscopy; **b** a plain computed tomography scan performed the following day.

**Fig. 3:**
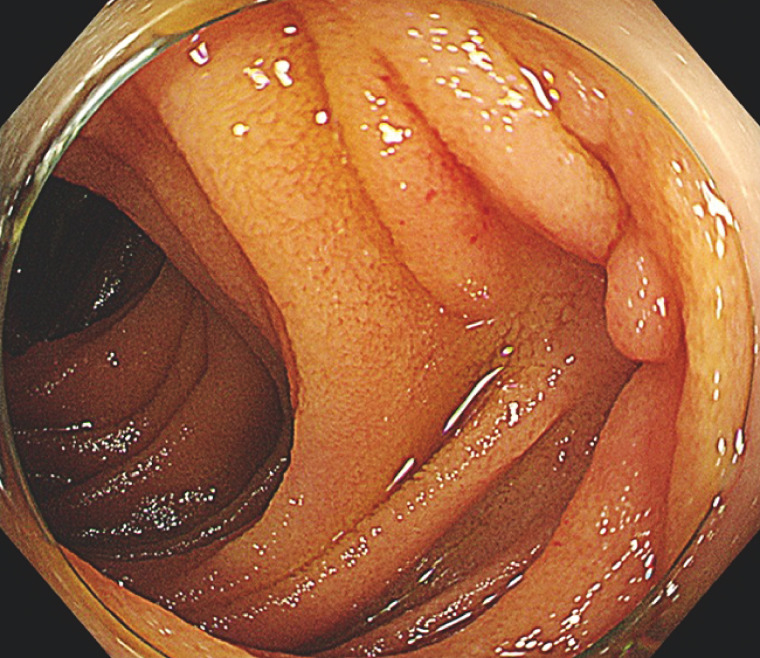
Image during follow-up endoscopy, 1 year later, showing the obliterated varices.

To the best of our knowledge, this is the first report of EUS-guided vascular intervention for pancreaticojejunal variceal bleeding. This procedure will be one of the options in future.

Endoscopy_UCTN_Code_TTT_1AS_2AG

## Notes

Endoscopy E-Videos https://eref.thieme.de/e-videos
*E-Videos* is an open access online section of the journal *Endoscopy* , reporting on interesting cases and new techniques in gastroenterological endoscopy. All papers include a high-quality video and are published with a Creative Commons CC-BY license. Endoscopy E-Videos qualify for HINARI discounts and waivers and eligibility is automatically checked during the submission process. We grant 100% waivers to articles whose corresponding authors are based in Group A countries and 50% waivers to those who are based in Group B countries as classified by Research4Life (see: https://www.research4life.org/access/eligibility/ ). This section has its own submission website at https://mc.manuscriptcentral.com/e-videos .
